# Methodological approach for determining the Minimal Important Difference and Minimal Important Change scores for the European Organisation for Research and Treatment of Cancer Head and Neck Cancer Module (EORTC QLQ-HN43) exemplified by the Swallowing scale

**DOI:** 10.1007/s11136-021-02939-6

**Published:** 2021-07-16

**Authors:** Susanne Singer, Eva Hammerlid, Iwona M. Tomaszewska, Cecilie Delphin Amdal, Kristin Bjordal, Bente Brokstad Herlofson, Marcos Santos, Joaquim Castro Silva, Hisham Mehanna, Amy Fullerton, Christine Brannan, Loreto Fernandez Gonzalez, Johanna Inhestern, Monica Pinto, Juan I. Arraras, Noam Yarom, Pierluigi Bonomo, Ingo Baumann, Razvan Galalae, Ourania Nicolatou-Galitis, Naomi Kiyota, Judith Raber-Durlacher, Dina Salem, Alexander Fabian, Andreas Boehm, Sanja Krejovic-Trivic, Wei-Chu Chie, Katherine Taylor, Christian Simon, Lisa Licitra, Allen C. Sherman, the EORTC Quality of Life Group and the EORTC Head and Neck Cancer Group

**Affiliations:** 1 Division of Epidemiology and Health Services Research, Institute of Medical Biostatistics, Epidemiology, and Informatics (IMBEI), University Medical Centre of Johannes Gutenberg University, Obere Zahlbacher Straße 69, 55131 Mainz, Germany; 2 Department of Otorhinolaryngology-Head and Neck Surgery, Institute of Clinical Sciences, Sahlgrenska Academy At University of Gothenburg, Sahlgrenska University Hospital, Gothenburg, Sweden; 3 Department of Medical Didactics, Jagiellonian University Medical College, Krakow, Poland; 4 Department of Oncology, Oslo University Hospital, Oslo, Norway; 5 Department of Research Support Services, Oslo University Hospital, Oslo, Norway; 6 Department of Oral Surgery and Oral Medicine, University of Oslo, Oslo, Norway; 7 Division for Head, Neck and Reconstructive Surgery, Department of Otorhinolaryngology - Head and Neck Surgery, Oslo University Hospital, Oslo, Norway; 8 Radiation Oncology Department, Grupo CONFIAR, Goiania, GO Brazil; 9 Department of Otolaryngology, Head and Neck Surgery, Instituto Português de Oncologia Francisco Gentil Do Porto, Porto, Portugal; 10 Institute of Head and Neck Studies and Education, University of Birmingham, Birmingham, UK; 11 Department of Communication Sciences and Disorders, Brooks Rehabilitation College of Healthcare Sciences, Jacksonville University, Jacksonville, FL USA; 12 Lynda Jackson Macmillan Centre, East & North Hertfordshire NHS Trust Incorporating Mount Vernon Cancer Centre, Northwood, UK; 13 Instituto Oncologico Fundación Arturo Lopez Perez, Santiago, Chile; 14 Department of Otorhinolaryngology, Oberhavelkliniken, Hennigsdorf, Germany; 15 Strategic Health Services Department, Istituto Nazionale Tumori –IRCCS- Fondazione G. Pascale, Napoli, Italy; 16 Oncology Departments, Complejo Hospitalario de Navarra, Pamplona, Spain; 17 Oral Medicine Unit, Sheba Medical Center, Tel-Hashomer, Israel; 18 School of Dental Medicine, Tel Aviv University, Tel Aviv, Israel; 19 Radiation Oncology Department, Azienda Ospedaliero Universitaria Careggi, Florence, Italy; 20 Department of Otolaryngology, Head and Neck Surgery, University of Heidelberg, Heidelberg, Germany; 21 MedAustron, Vienna, Austria; 22 Dental Oncology Unit, Clinic of Hospital Dentistry, School of Dentistry, National and Kapodistrian University of Athens, Athens, Greece; 23 Department of Medical Oncology and Hematology, Kobe University Hospital Cancer Center, Kobe, Japan; 24 Department of Oral and Maxillofacial Surgery, Amsterdam University Medical Center, University of Amsterdam, Amsterdam, The Netherlands; 25 Department of Oral Medicine ACTA, University of Amsterdam and Vrije Universiteit, Amsterdam, The Netherlands; 26 Department of Medical Oncology, Ain Shams-University, Cairo, Egypt; 27 Department of Radiation Therapy, University Hospital Schleswig-Holstein, Kiel, Germany; 28 Department of Otolaryngology Head and Neck Surgery, St. Georg Hospital, Leipzig, Germany; 29 Clinic of Otorhinolaryngology and Maxillofacial Surgery, Faculty of Medicine, University of Belgrade, Belgrade, Serbia; 30 Institute of Epidemiology and Preventive Medicine, College of Public Health, National Taiwan University, Taiwan, Taiwan; 31 Department of Otolaryngology, Lausanne, Switzerland; 32 Department of Medical Oncology, Fondazione IRCCS Istituto Nazionale Dei Tumori, Milano, Italy; 33 Behavioral Medicine Division, Winthrop P. Rockefeller Cancer Institute, University of Arkansas for Medical Sciences, Little Rock, USA

**Keywords:** Minimal important difference, Minimal important change, MCID, Clinical significance, Subjective significance, EORTC QLQ-HN43

## Abstract

**Purpose:**

The aim of this study was to explore what methods should be used to determine the minimal important difference (MID) and minimal important change (MIC) in scores for the European Organisation for Research and Treatment of Cancer Head and Neck Cancer Module, the EORTC QLQ-HN43.

**Methods:**

In an international multi-centre study, patients with head and neck cancer completed the EORTC QLQ-HN43 before the onset of treatment (t1), three months after baseline (t2), and six months after baseline (t3). The methods explored for determining the MID were: (1) group comparisons based on performance status; (2) 0.5 and 0.3 standard deviation and standard error of the mean. The methods examined for the MIC were patients' subjective change ratings and receiver-operating characteristics (ROC) curves, predictive modelling, standard deviation, and standard error of the mean. The EORTC QLQ-HN43 Swallowing scale was used to investigate these methods.

**Results:**

From 28 hospitals in 18 countries, 503 patients participated. Correlations with the performance status were |*r*|< 0.4 in 17 out of 19 scales; hence, performance status was regarded as an unsuitable anchor. The ROC approach yielded an implausible MIC and was also discarded. The remaining approaches worked well and delivered MID values ranging from 10 to 14; the MIC for deterioration ranged from 8 to 16 and the MIC for improvement from − 3 to − 14.

**Conclusions:**

For determining MIDs of the remaining scales of the EORTC QLQ-HN43, we will omit comparisons of groups based on the Karnofsky Performance Score. Other external anchors are needed instead. Distribution-based methods worked well and will be applied as a starting strategy for analyses. For the calculation of MICs, subjective change ratings, predictive modelling, and standard-deviation based approaches are suitable methods whereas ROC analyses seem to be inappropriate.

**Supplementary Information:**

The online version of this article (10.1007/s11136-021-02939-6) contains supplementary material, which is available to authorized users.

## Background

Quality of Life (QoL) domains are usually reported in terms of scores. In order to assess the effects of a new drug or intervention, researchers must determine the minimal difference in these scores deemed clinically important. Only by knowing that, can they calculate the sample size for a trial and interpret which results are clinically meaningful.

Likewise, clinicians, patients, and policy-makers need to know what changes in QoL scores over time or differences in scores between groups are clinically relevant. If, say, we have a scale with a potential range of 0–100, and a patient had a score of 87 before surgery and 80 afterwards, would the difference be clinically relevant?

The difficulty lies in (a) how best to define these concepts and (b) how to measure them empirically. Numerous terms are used to describe the issue at hand—minimal important difference, minimal detectable change, clinical significance, etc. [1]. We will use the terms minimal important difference (MID) and minimal important change (MIC), the MID being the minimal difference in QoL *between patient groups* that is clinically relevant and the MIC being the minimal change in QoL *over time* that is clinically relevant [2].

A familiar definition for MID is the least difference that would lead to a change in treatment [1, 3, 4], while the MIC is defined as the minimal difference over time considered relevant by the patient [5]. Both can be measured using so called anchor-based approaches (which map QoL scores onto an external indicator) or distribution-based approaches (which rely on statistical criteria). Several papers summarise and discuss these approaches and the various methods for deriving estimates [1, 6–9]. There is no "gold-standard" method for estimating MID or MIC. Distribution-based methods alone have often been found insufficient [10] because they do not directly capture the patient’s or clinician’s perspective regarding the meaning of scores, and this should be resolved by combining them with anchor-based approaches. A further recommendation is to report a range of numbers instead of a single one, since different methods may yield different estimates [1].

A difference of 10 points is often assumed to be the appropriate MID and MIC for the EORTC QLQ-C30 (European Organisation for Research and Treatment of Cancer Quality of Life Core Questionnaire), based on the work of Osoba [5]. Cocks et al. recommended using scale-specific MICs [11]. Recently, the EORTC Quality of Life Group performed analyses of previous EORTC trials to define various MICs for the EORTC QLQ-C30 scales [12]. The MIC definitions were obtained by other authors carrying out observational studies [13, 14] or using existing data from past clinical trials [15]. For the EORTC disease-specific modules (as opposed to the core instrument) initial studies investigating MIDs or MICs have been published [16, 17]. It is our aim to calculate MID and MIC estimates for the recently updated head and neck module, the EORTC QLQ-HN43 [18–20]. As there is no gold standard method for calculating MID and MIC, we first developed a methodological approach, exploring various methods, the results of which are presented in this paper.

The research questions of the current study were: 1. What methods should we use to determine the clinically relevant minimal score differences of the EORTC QLQ-HN43 scales between patient groups (the MID)? 2. What methods should we use to determine the clinically relevant minimal changes in score over time for the EORTC QLQ-HN43 scales (the MIC)?

In the current paper, we focus on the Swallowing scale, in view of the importance of swallowing difficulties for patients with head and neck cancer and its wide use in clinical studies [21]. We anticipated that these efforts would provide a useful model for approaching the determination of MIDs/MICs for the other scales in the EORTC QLQ-HN43 module.

## Methods

### Study design

In an international, multi-centre prospective validation study of the updated EORTC head and neck cancer module [18], patients with head and neck cancer under active treatment (Group 1) completed a questionnaire at the following time points: before the onset of treatment (t1), three months after baseline (t2), and six months after baseline (t3). Based on previous studies [22–27] and on clinical experience, we assumed Quality of Life would deteriorate for most patients between t1 and t2 and would somewhat improve between t2 and t3. In the validation study, there was also a group of head and neck cancer post-treatment survivors (Group 2) included to determine test–retest reliability. For the determination of MID and MIC presented in this paper, we used the data of Group 1 only.

### Inclusion criteria, exclusion criteria, and data collection

Patients with the following ICD-10 codes were included: larynx (C32), lip (C00), oral cavity (C01-06), salivary glands (C07-08), oro-hypopharynx (C09-10, C12-14), nasopharynx (C11), nasal cavity (C30), nasal sinuses (C31), sarcoma in the head and neck region (C49), and lymph node metastases from unknown primary in the head and neck area (C77, C80.0). We did not include patients with tumours of the eyes, orbit, thyroid, skin (even if in the head and neck area), or lymphomas in the head and neck region. Additional inclusion criteria were sufficient language proficiency and sufficient cognitive functioning (assessments made by study coordinator), aged 18 years or over, and written informed consent.

Upon admission to the hospital or clinic, eligible patients received an invitation to participate in the study, and oral and written information in accordance with ethical and governance requirements of each participating centre. All sites obtained ethical approval in accordance with regional and national requirements. Patients were given time to consider the study and ask any questions before consenting and participating.

### Instruments

The EORTC QLQ-C30 [28] and the EORTC QLQ-HN43 [18, 19] questionnaires were administered at all three time points.

At t2 and t3, a subset of participants also completed the Subjective Significance Questionnaire (SSQ) [5]. In the SSQ, patients were asked to rate the extent that their QoL had changed (improved or worsened) in the domains swallowing, speech, dry mouth, and global quality of life compared to the previous time point. The first three domains were chosen as they were previously rated as having the highest priority by patients with head and neck cancer [20] and global quality of life was included because of its general applicability. The response options for each of these items ranged from very much worse to very much better on a 7-point Likert scale. Consistent with the literature, the options "a little worse" and "a little better" defined the MIC from the patient's perspective, since these categories represent minimal change [29]. The current analyses included the Swallowing item, since this was most relevant for the EORTC QLQ-HN43 Swallowing scores.

Information on the patient's gender, age, education, tumour site, tumour stage, Karnofsky Performance Score (KPS), and treatment received was documented on a Case Report Form by study staff.

### Analysis

The statistical analysis plan was developed based on information from published papers and experiences from research clinicians involved in the study. After discussions in the group, we decided to employ a variety of methods to determine the MID and MIC in order to examine their applicability for the EORTC QLQ-HN43 scales using the Swallowing scale as an example. The results of this should serve as a decision basis for what methods to use when analysing the MID and MIC for all the other EORTC QLQ-HN43 scales. The Swallowing scale was used because it was applied most often in previous trials and clinical studies according to a systematic review [21].

#### Descriptive analyses

The sample for the MID and MIC analyses comprised patients under active treatment who participated at least twice. The frequencies and percentages of the following variables were calculated: gender, age, education, tumour site, UICC tumour stage, Karnofsky Performance Score (KPS), and treatment received (as documented at t2).

For the EORTC QLQ-HN43 Swallowing scale, the mean change (delta—∆) and its standard deviation, minimum, and maximum were calculated for changes between t1 and t2 and between t2 and t3.

#### Methods for determining the Minimal Important Difference (MID)

##### 1. Anchor-based approach

The assumption was that patients differ clinically when they have a KPS of 60 (requires some assistance but able to care for most of own needs) vs. 70 (cares for self, unable to carry on normal activity or do active work) and when they have a KPS of 70 vs. 80 (normal activity with effort), since these are often the thresholds for participation in clinical trials and treatment recommendations. However, it was unclear whether KPS correlates with the various domains of the EORTC QLQ-HN43 questionnaire, which is necessary if it is to work as a suitable anchor. The group decided, therefore, to calculate the Spearman correlation coefficients for KPS at t2 with the EORTC QLQ-HN43 scales at t2. If the correlation coefficient with KPS was |*r*|≥ 0.40, the following calculations were planned: mean difference for the EORTC QLQ-HN43 scale score in patients with KPS 60 vs. 70 (at t2), mean difference for the EORTC QLQ-HN43 scale score in patients with KPS 70 vs. 80 (at t2). If the correlation coefficient was |*r*|< 0.40, we considered that it was not a suitable anchor for this scale in this population [30]. The calculation of the Spearman correlation coefficients for KPS with the EORTC QLQ-HN43 scales were repeated for t1 to investigate robustness of the results.

##### 2. Distribution-based approach

We calculated the 0.5 and the 0.3 standard deviation [7] and the standard error of measurement (SEM) of the Swallowing scale score at t2. The SEM was defined as follows: SEM = SD * square root of (1-Cronbach’s Alpha), which gives the measurement error for an individual measurement (i.e. at patient-level). The values for Cronbach’s alpha are published elsewhere [18]. For the Swallowing scale, the Cronbach’s alpha was 0.85 at t2.

#### Methods for determining the Minimal important change (MIC)

##### 1. Anchor-based approach

We calculated the mean delta of the Swallowing scale scores for those patients who reported their swallowing had changed “a little” for changes between t1 and t2, as well as between t2 and t3. Calculations were made separately for patients with improved and deteriorated swallowing.

In addition, we used Receiver Operating Characteristics (ROC) curves, as suggested by Kvam et al. [13]. The procedure was as follows:

- We created groups of patients with improved, unchanged, and deteriorated swallowing, using responses for the Swallowing change item from the SSQ. Responses of “very much worse,” “moderately worse,” and “a little worse” were classified together as “deteriorated”, and responses of “very much better,” “moderately better,” and “a little better” were classified together as “improved”. Patients responding “about the same” were classified as “unchanged”.
- Based on step a, two dichotomous variables were created for the SSQ swallowing anchor: improved vs. not improved (with “unchanged” and “deteriorated” considered as “not improved”) and deteriorated vs. not deteriorated (with “unchanged” and “improved” considered as “not deteriorated”).
- The area under the curve (AUC) was calculated separately for deterioration between t1 and t2, because most patients were expected to experience a worsening of functioning during the treatment period when toxicities are pronounced, and improvement between t2 and t3, because most participants were expected to report gains in functioning during the immediate post-treatment period. The cut-off point with the highest Youden-Index (sensitivity + specificity-1) was considered to be the MIC [31].

Lastly, we applied predictive modelling to obtain MIC estimates as suggested by Terluin [32]. Here, the MIC is defined as (ln(odds-pre)—intercept)/regression coefficient.

##### 2. Distribution-based approach

We calculated the 0.3 and 0.5 standard deviation as well as the SEM of the delta for Swallowing scores between t1 and t2 to determine the MIC for deterioration and the 0.3 and 0.5 standard deviation as well as SEM of the delta in Swallowing scores between t2 and t3 to determine the MIC for improvement.

## Results

### Sample

**Fig. 1:**
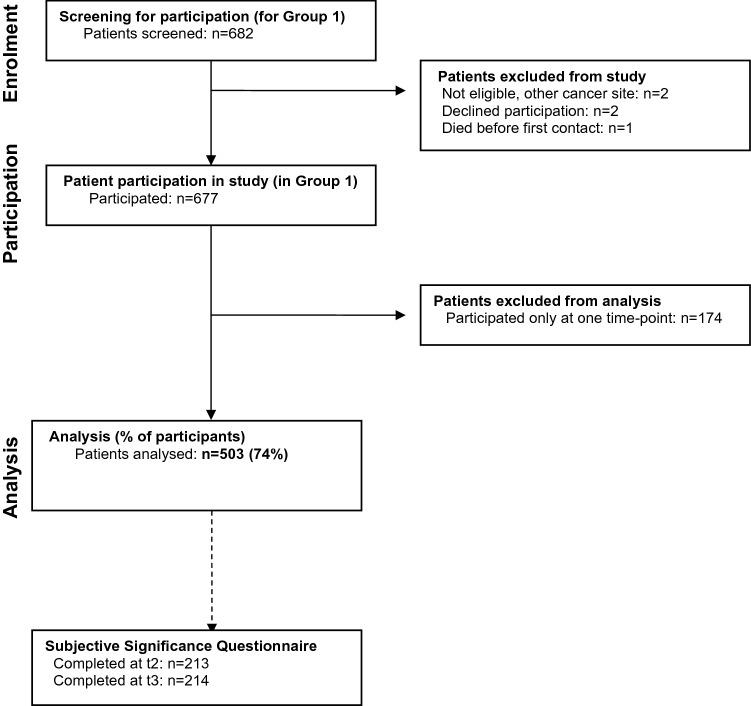
Patient flow through the study

From 28 treatment centres in 18 countries, 812 patients were enrolled into the validation study, of which 677 were in Group 1 (Fig. 1). Of these, 503 participated at more than one time point, and their data were used for the MID and MIC analyses; 108 participated twice and 395 thrice. The patient characteristics are displayed in Table 1. KPS at t1 ranged from 40 to 100 (mean = 89, skewness = − 1.1), at t2 from 20 to 100 (mean = 82, skewness = − 0.8), and at t3 from 10 to 100 (mean = 84, skewness = − 1.4).

**Fig. 2:**
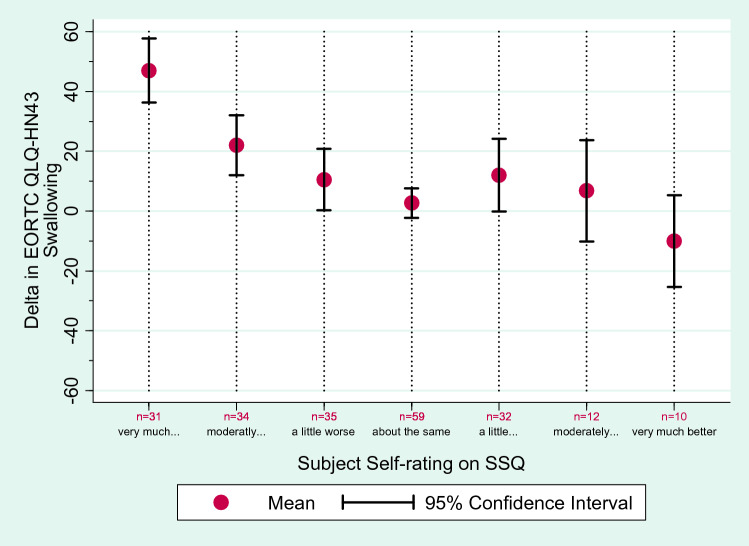
Subjective changes in swallowing between t1 and t2, measured with the Subjective Significance Questionnaire (SSQ), and the corresponding delta (mean and 95% confidence interval) in the Swallowing Scale of the EORTC QLQ-HN43

**Table 1:** Patient characteristics (*n* = 503)

	*N* (%)	Percent (%)
Cancer site		
Larynx	81	16
Hypopharynx	44	9
Oropharynx	173	34
Oral cavity	151	30
Parotid gland	22	4
Nasal cavity and sinuses	17	3
Unknown primary	13	3
Missing information	2	0
Cancer stage		
I	59	12
II	90	18
III	93	18
IV	249	50
Missing information	12	2
Karnofsky Performance Score (at baseline)		
40	2	0.4
50	1	0.2
60	11	2
70	40	8
80	87	17
90	174	35
100	180	36
Missing information	8	2
Treatment		
Surgery alone	95	1
Radiotherapy alone	86	17
Chemotherapy alone	11	2
Radiochemotherapy without surgery	136	27
Radiochemotherapy with surgery	74	15
Surgery and chemotherapy	6	1
Surgery and radiotherapy	68	14
Other	27	5
Gender		
Male	365	73
Female	134	27
Missing information	4	1
Age		
< 50 years	50	10
50–59 years	134	27
60–69 years	178	35
70–79 years	113	22
≥ 80 years	27	5
Missing information	1	0
Education		
< 10 years	157	31
10 years	83	17
> 10 years	223	44
Missing information	40	8

There was no evidence that age, gender, education, tumour site, and tumour stage differed between patients who participated just once to those who participated more than once (data not shown).

### Mean change in the Swallowing scale score

On average, patients reported increased difficulties with swallowing during the treatment period, followed by partial recovery in the acute post-treatment phase. Between t1 and t2, swallowing problems increased by approximately 13 points on average (SD = 31, range: − 100– + 100). Between t2 and t3, the mean delta was − 8 (SD = 25, range: − 92– +100).

### Minimal important difference (MID)

#### Anchor-based approach

The correlation of the KPS with the EORTC QLQ-HN43 Swallowing scale at t2 was − 0.36, which fell below the required threshold of 0.40. More broadly, the correlations between the KPS and the EORTC QLQ-HN43 scales ranged from − 0.12 (Neurological Problems) to − 0.42 (Social Contact). Of all the 19 scales, only two (Social Contact and Social Eating) correlated |*r*| ≥ 0.40 with the KPS. The correlation of the KPS with the EORTC QLQ-HN43 Swallowing scale at t1 gave similar results (see Supplemental Material, eTable 1 for details). Based on these results, it was concluded that the KPS is not a valid external anchor for group comparisons for this module and no further analyses with this approach were performed.

#### Distribution-based approach

The 0.5 and 0.3 standard deviation estimates of the Swallowing scale at t2 were 14.3 and 9.5, respectively. The standard error of measurement (SEM) of the Swallowing scale was 11.

### Minimal important change (MIC)

#### Anchor-based approach

##### Using patient ratings of "a little change" in the SSQ

A total of 213 patients completed the SSQ at t2, and 214 at t3 (Table 2). At t2, 35 patients reported that their swallowing had worsened a little compared to t1. The respective mean delta in EORTC QLQ-HN43 Swallowing was 11 points (95% CI 0; 21). Thirty-two patients reported their swallowing had improved a little at t2 on the SSQ; the mean delta was 12 (95% CI 0; 24). The correlation coefficient between the SSQ Swallowing score and the delta in EORTC QLQ-HN43 Swallowing was *r* = − 0.42 (correlation of SSQ with Swallowing score at t1 was *r* = 0.001 and with Swallowing score at t2 *r* = − 0.46). Notably, the mean delta in EORTC QLQ-HN43 Swallowing scores for the improved group was a deterioration of 12 points (95% CI 0; 24), meaning that their scores in the Swallowing scale *worsened* on average by 12 points even though all these patients subjectively reported improved swallowing function. As this was a counterintuitive finding, we looked at the number of patients with positive and negative delta in more detail, to find out whether the result was due to an outlier. Of those 32 patients who said their swallowing had improved a little on the SSQ, 11 had indeed a lower (better) score in the Swallowing scale at t2 compared to t1, but 16 had a higher score, indicating more swallowing difficulty at t2 compared to t1. The remaining patients had the same Swallowing score at t1 and t2. This implies that the results were not due to few outliers. (Fig. 2).

**Table 2:** Subjective changes in swallowing, measured with the SSQ, and the corresponding delta in the Swallowing Scale of the EORTC QLQ-HN43

Patients reporting their swallowing is…	t1–t2					t2–t3				
	Delta in Swallowing					Delta in Swallowing				
	*N*	Percent (%)	Mean	Min	Max	*N*	Percent (%)	Mean	Min	Max
Very much worse	31	15	47	− 17	100	9	4	27	− 33	100
Moderately worse	34	16	22	− 100	92	10	5	9	− 50	75
A little worse	35	16	11	− 58	83	16	7	18	− 33	75
About the same	59	28	3	− 50	58	71	33	− 2	− 58	67
A little better	32	15	12	− 50	83	45	21	− 14	− 58	42
Moderately better	12	6	7	− 25	58	34	16	− 12	− 83	25
Very much better	10	5	− 10	− 33	42	29	14	− 17	− 50	8
All patients	213		13	− 100	100	214		− 8	− 83	100

A positive change score (delta) implies that the problems with swallowing have increased (i.e., quality of life is worse); negative scores imply fewer problems (i.e., quality of life is better)

*SSQ* Subjective Significance Questionnaire, *EORTC QLQ-HN43* head and neck cancer specific module, *t* time

At t3, 16 patients reported a little worse swallowing on the SSQ; the corresponding mean delta in the EORTC QLQ-HN43 Swallowing scale was 18 points (95% CI 4; 32). At t3, 45 patients said their swallowing was a little better on the SSQ, and the mean delta on the EORTC QLQ-HN43 Swallowing scale was -14 points (95% CI − 21; − 7), reflecting the expected improvement. The correlation coefficient between the SSQ Swallowing score at t3 and the t2-t3-delta in EORTC QLQ-HN43 Swallowing was *r* = − 0.41 (correlation of SSQ with Swallowing score at t2 was *r* = 0.03 and with Swallowing score at t3 *r* = − 0.39).

##### Using ROC curves

The ROC curve-derived MIC for deterioration was 8 (Table 3 and Supplemental Material for details), derived from patients who reported a deterioration in Swallowing between t1 and t2 in the SSQ. The corresponding AUC was 0.73 and the Youden index was 0.41. The AUC value suggests that this analysis was able to discriminate between anchor groupings better than chance alone during the treatment period.

**Table 3:** Minimal important change scores according to the ROC analyses

Patients say their swallowing is	*N*	Cutpoint	Sensitivity	Specificity	Youden-Index	Area under the curve (95% confidence interval)		
Deteriorated (between t1 and t2)	100 (of 213)	8	71%	70%	0.41	0.73	(0.66;	0.80)
Improved (between t2 and t3)	108 (of 214)	− 83	100%	0%	0.00	0.29	(0.22;	0.36)

*ROC* receiver-operating characteristics, *t* time

Positive scores indicate more problems with swallowing

The MIC for improvement was − 83, based on data from patients who reported improvement in Swallowing between t2 and t3, which is not a plausible number. The AUC was 0.29 and the Youden index 0; i.e., both indicate poor performance of the model during the post-treatment period (see eFigure 1 in Supplemental Material for details). The calculations were repeated for improvement between t1 and t2 but the outcome remained the same (data not shown).

In both calculations, there were ties, which may have biased the estimates.

##### Using predictive modelling

The MIC derived from regression analysis was 14.6 for deterioration (odds_pre_ 0.89, regression coefficient = 0.03, 95% CI: 0.02 to 0.04, intercept = − 0.51) and − 3.1 for improvement (odds_pre_ 1.02, regression coefficient = -0.03, 95% CI: − 0.05 to − 0.02, intercept = − 0.09).

#### Distribution-based approach

The standard deviation of the delta in Swallowing scale score between t1 and t2 was 32 points. The corresponding MICs for deterioration were 10 (0.3 SD), 16 (0.5 SD), and 12 (SEM) points.

The standard deviation of the delta in Swallowing between t2 and t3 was 25 points. The MICs for improvement were, therefore, 8 (0.3 SD), 12 (0.5. SD), and 10 (SEM) points.

### Results of various approaches combined

The MID for the Swallowing scale ranged from 10 to 14, the MIC for deterioration from 8 to 16 and the MIC for improvement from − 3 to − 14 (Table 4).

**Table 4:** Minimal important difference (MID) and change (MIC) scores for the EORTC QLQ-HN43 Swallowing Scale, derived by various approaches

	MID	MIC	
		Deterioration (t1 to t2)	Improvement (t2 to 3)
Anchor-based	Mean difference of x at t2 in patients with KPS 60 vs. KPS 70 at t2	Mean delta in patients who say that their swallowing is “a little worse”	Mean delta in patients who say that their swallowing is “a little better”
	*Discarded because of poor correlation*	11	− 14
	Mean difference of x at t2 in patients with KPS 70 vs. KPS 80 at t2	ROC derived cut-point	ROC derived cut-point
	*Discarded because of poor correlation*	8	*Discarded because of poor AUC*
		Based on predictive regression modelling	Based on predictive regression modelling
		15	− 3
Distribution-based	0.5 SD of Swallowing at t2	0.5 SD of delta in Swallowing	0.5 SD of delta in Swallowing
	14	16	− 12
	0.3 SD of Swallowing at t2	0.5 SD of delta in Swallowing	0.5 SD of delta in Swallowing
	10	10	− 8
	SEM at t2	SEM of delta in Swallowing	SEM of delta in Swallowing
	11	12	− 10

*SD* standard deviation, *SEM* standard error of the mean, *ROC* receiver-operating characteristics, *AUC* area under the curve, *t* time, *KPS* Karnofsky Performance Score

## Discussion

In this study, we examined which methods would be useful to determine the MID and MIC for the head and neck cancer module of the EORTC questionnaire. Both distribution- and anchor-based approaches were used and applied to the Swallowing scale because this is an important domain of QoL in head and neck cancer patients, and the corresponding scale in the EORTC instrument is most often used in clinical studies [21]. The aim was to explore which of the methods can be used later on for determining the MIC for all scales of the EORTC QLQ-HN43.

The various results were presented side by side (anchor-based vs. distribution-based; MID vs. MIC; results for deterioration vs. improvement). Although clinicians often prefer integration of results into single MID and MIC values, it is important first to understand the variety of findings and explore the applicability of the various approaches. It is also essential to keep in mind that the various estimates found in our study are based on conceptually different approaches (for example, the criterion for change can be defined by the patient or by external anchors). Researchers must determine which concept is most appropriate for their study.

The findings show that the anchor-based approach was ineffective in defining minimal important differences between patient groups (the MID) as the only external anchor available was the Karnofsky Performance Score, and correlations with it were poor, according to our predefined thresholds. This result highlights the importance of verifying instead of assuming that potential anchors have meaningful associations with the target QoL measure. A recent review [9] reported that roughly a quarter of oncology investigations seeking to determine anchor-based MIDs for patient-reported outcome measures neglected to verify these correlations. In the current study, the modest correlation between performance status and the EORTC QLQ-HN43 Swallowing scale removed a convenient anchor; on the other hand, these results seem to bolster the discriminant validity of the EORTC QLQ-HN43 Swallowing scale and the other domains, since they were initially developed largely because clinician-rated performance measures were deemed inadequate for capturing the richness and nuances of patients’ experiences. Future studies could explore whether other external anchors are more suitable; using the current example of the Swallowing scale, tools that objectively assess swallow function or use of feeding tube, such as the Functional Oral Intake Scale [33], penetration-aspiration score [34] or Dynamic Grade of Imaging Toxicity [35] might be useful; for the Social Eating scale a subjective score of functional behaviour (for example, frequency of patient eating out) such as the MD Anderson Dysphagia Inventory [36] or Mann Assessment of Swallow Ability [37] might be used. It is likely that external anchors are scale-specific, i.e., they cannot be used to determine the MID for all scales.

Distribution-based approaches were applicable. The criteria of one-third and one-half standard deviation and standard error of the mean yielded MIDs between 9.5 and 14.3. The advantage of the standard error of the mean is that it is relatively independent from the sample size, as it is largely an attribute of the measure rather than a characteristic of the sample [2]. However, on their own, distribution-based methods are often considered suboptimal relative to those which are anchor-based as they are not intuitively understood by clinicians or patients and do not directly reflect patients’ perceptions of meaningful differences [2, 8]. So, what alternatives can be applied if we want to find group differences that are relevant to patients? Cocks et al. performed qualitative interviews with breast cancer patients and discovered that patients are able to interpret findings from published literature and give opinions about the significance of differences found between groups [38]. Similarly, Sully et al. used qualitative interviews to explore meaningful QoL score changes among multiple myeloma patients [16]. This suggests patients' opinions can work as an external anchor. Although this is an interesting approach, it requires additional data collection and careful interviewing; calculations cannot simply be performed using existing data which is why we could not use it.

The anchor-based approach using subjective patient ratings for determining minimal clinically relevant changes over time (the MIC) yielded—in part—useable results. Problems occurred when we applied ROC methodology, especially when investigating *improvement* of quality of life; patients sometimes rated their quality of life retrospectively as improved although their module scores had actually worsened during that interval (this phenomenon was observed in both time intervals). This was an interesting observation as both measures, the SSQ and the EORTC QLQ-HN43, were completed by patients themselves. Patients were asked in the EORTC module to assess their current ability to swallow (solid food, pureed food, liquids, etc.), whereas in the SSQ they were asked to make a retrospective judgment on their changes in swallowing compared to the previous measurement 3 months before. Obviously, the change score required more cognitive and emotional processing: patients were asked to make a judgement on the status of their current condition, recall the previous status of their condition, and make comparisons and a judgement of change between the two. It is likely that (dis)satisfaction with the changes may additionally influence the latter. Satisfaction itself may be viewed as comprising two components: the expectations we have and the evaluation of the situation. This can lead to the so-called satisfaction paradox: if patients expect little improvement, they may be more satisfied with small improvements than if they had expected things to be much better, and vice versa [39]. In this case, perhaps some patients experienced less deterioration in swallowing than they had anticipated or possibly an adaptive sensory response to physiological motoric decline. Other processes that most likely play a role here are response shift and recall bias [6, 40, 41].

This finding emphasises there is no ‘one size fits all’ approach for determining MIC even for patient global ratings of change. Therefore, the conclusion of our study group was to continue using a variety of concurrent approaches – distribution and anchor-based. As we move forward in future studies to determine MICs and MIDs of the other scales in the EORTC QLQ-HN43, we plan to omit ROC analyses and comparisons of groups based on the Karnofsky Performance Score and apply all the other methods. It is hoped that additional investigators will be able to evaluate additional clinical anchors. It should be noted though that the results of the methods are particular to this specific study. Although not viable for the current dataset, ROC analyses were suitable methods to estimate the MIC in other studies [13].

While developing the statistical analysis plan, we realised that many decisions needed to be taken prior to knowing the results and the difficulties this would entail. However, we also wanted to avoid "fishing for the best results". Consequently, we agreed to be decisive in certain aspects beforehand, and more explorative in others. For example, based on previous literature [22–27], we assumed that swallowing deteriorates between the time before treatment starts and three months later and we assumed improvement of swallowing between three and six months after baseline. We therefore decided to compare scores with "a little change" in the patient ratings between these two time spans and investigated the MIC for deterioration between t1 and t2 and the MIC for improvement between t2 and t3. However, was this a good decision? There was indeed an average deterioration of EORTC QLQ-HN43 Swallowing scores between t1 and t2 and an improvement between t2 and t3 on a group-level, but there were also some patients where the reverse was true. This might be related to improved symptom relief including pain medication. Moreover, data were considerably heterogeneous which could have contributed to the pattern of results that we observed.

Another point for discussion is that the mean change score on the EORTC QLQ-HN43 Swallowing Scale was not zero for "no change" on the anchor. In future studies, a calibration could be used in such situations, i.e., taking the difference between mean changes on the EORTC QLQ-HN43 Scale of interest between adjacent categories of the anchor measure.

Another potential limitation is that we had decided a priori to calculate the distribution-based values for data at t2, not at t1 or t3. We did so because the time-point matched with one that is frequently used in clinical trials. We did not calculate it for all time-points because we wanted to establish a method that could be applied to all scales of the module and restrict the number of possible MID and MIC values for one scale to a reasonable amount. Failure to do so could potentially confuse clinicians and consequently let them return to the simpler 10-point rule [5] or the 16% of the range-rule [8]. However, concentrating on only one time point bears risks. For example, if Cronbach’s alpha of the instrument differs following treatment (t2) in contrast to before (t1), then these SEM-based estimates differ as well. In our study, the differences in reliability were luckily very small (Cronbach’s alpha was 0.83 at t1, 0.85 at t2 and 0.85 at t3).

A further point discussed in our group was the difficulty encountered in trying to determine MIC and MID, and we consider thresholds [42, 43] as a potential alternative. However, we decided to continue determining MIDs and MICs because of their importance not only for researchers and clinicians but also for regulatory bodies.

## Conclusions

In summary, the current study used a variety of anchor- and distribution-based approaches to examining MIDs and MICs for Swallowing scores in a newly refined QoL instrument, the EORTC QLQ-HN43. The investigation drew on a large, international database encompassing repeated assessments completed by over 500 patients from 28 treatment centres around the world. To develop a feasible model, we focused on impaired swallowing, a domain of QoL that is of direct importance to head and neck cancer patients and clinicians. Findings illustrate some of the challenges of obtaining appropriate clinical anchors. Nonetheless, the estimates generated may help clinicians and investigators to interpret the meaning of EORTC QLQ-HN43 Swallowing scores and plan investigations. Results identified a number of strategies that appear to be useful in generating MIDs and MICs for this instrument, and we look forward to further examining their value with respect to additional scales on the module.

## Supplementary Information

Below is the link to the electronic supplementary material.

(DOCX 508 kb)

## Data Availability

The data of this study are stored in the EORTC data repository and can be accessed by other researchers.

## Code availability

The software used for this study was STATA 12.1 (StataCorp LP, Texas, USA).

## References

[1] King MT (2011). A point of minimal important difference (MID): A critique of terminology and methods. Expert Review of Pharmacoeconomics & Outcomes Research.

[2] Crosby RD, Kolotkin RL, Williams GR (2003). Defining clinically meaningful change in health-related quality of life. Journal of Clinical Epidemiology.

[3] Jaeschke R, Singer J, Guyatt GH (1989). Measurement of health-status: Ascertaining the minimal clinically important difference. Controlled Clinical Trials.

[4] Sloan JA, Cella D, Frost M, Guyatt GH, Sprangers M, Symonds T (2002). Assessing clinical significance in measuring oncology patient quality of life: Introduction to the symposium, content overview, and definition of terms. Mayo Clinic Proceedings.

[5] Osoba D, Rodrigues G, Myles J, Zee B, Pater J (1998). Interpreting the significance of changes in health-related quality-of-life scores. Journal of Clinical Oncology.

[6] Cella D, Hahn EA, Dineen K (2002). Meaningful change in cancer-specific quality of life scores: Differences between improvement and worsening. Quality of Life Research.

[7] Ringash J, O'Sullivan B, Bezjak A, Redelmeier DA (2007). Interpreting clinically significant changes in patient-reported outcomes. Cancer.

[8] Sloan JA, Frost MH, Berzon R, Dueck A, Guyatt G, Moinpour C (2006). The clinical significance of quality of life assessments in oncology: A summary for clinicians. Supportive Care in Cancer.

[9] Ousmen A, Touraine C, Deliu N, Cottone F, Bonnetain F, Efficace F, Brédart A, Mollevi C, Anota A (2018). Distribution and anchor-based methods to determine the minimally important difference on patient-reported outcome questionnaires in oncology: A structured review. Health and Quality of Life Outcomes.

[10] Lemieux J, Beaton DE, Hogg-Johnson S, Bordeleau LJ, Goodwin PJ (2007). Three methods for minimally important difference: No relationship was found with the net proportion of patients improving. Journal of Clinical Epidemiology.

[11] Cocks K, King MT, Velikova G, de Castro G, St James MM, Fayers PM (2012). Evidence-based guidelines for interpreting change scores for the European Organisation for the Research and Treatment of Cancer Quality of Life Questionnaire Core 30. European Journal of Cancer.

[12] Musoro ZJ, Hamel JF, Ediebah DE, Cocks K, King MT, Groenvold M, Sprangers MA, Brandberg Y, Velikova G, Maringwa J, Flechtner HH (2018). Establishing anchor-based minimally important differences (MID) with the EORTC quality-of-life measures: A meta-analysis protocol. British Medical Journal Open.

[13] Kvam AK, Fayers P, Wisloff F (2010). What changes in health-related quality of life matter to multiple myeloma patients? A prospective study. European Journal of Haematology.

[14] Bedard G, Zeng L, Zhang LY, Lauzon N, Holden L, Tsao M (2016). Minimal important differences in the EORTC QLQ-C15-PAL to determine meaningful change in palliative advanced cancer patients. Asia-Pacific Journal of Clinical Oncology.

[15] Raman S, Ding KY, Chow E, Meyer RM, van der Linden YM, Roos D (2018). Minimal clinically important differences in the EORTC QLQ-C30 and brief pain inventory in patients undergoing re-irradiation for painful bone metastases. Quality of Life Research.

[16] Sully K, Trigg A, Bonner N, Moreno-Koehler A, Trennery C, Shah N (2019). Estimation of minimally important differences and responder definitions for EORTC QLQ-MY20 scores in multiple myeloma patients. European Journal of Haematology.

[17] Reni M, Braverman J, Hendifar A, Li CP, Mercade TM, Oh DY (2019). Evaluation of minimal important difference (MID) for the European organisation for research and treatment of cancer (EORTC) pancreatic cancer module (PAN26) in patients with surgically resected pancreatic adenocarcinoma. Annals of Oncology.

[18] Singer S, Amdal CD, Hammerlid E, Tomaszewska IM, Silva JC, Mehanna H (2019). International validation of the revised European Organisation for Research and Treatment of Cancer Head and Neck Cancer Module, the EORTC QLQ-HN43: Phase IV. Head and Neck.

[19] Singer S, Araújo C, Arraras J, Baumann I, Boehm A, Herlofson BB (2015). Measuring quality of life in head and neck Cancer patients: Update of the EORTC QLQ-H&N Module Phase III. Head and Neck.

[20] Singer S, Arraras J, Baumann I, Boehm A, Chie WC, Galalae R (2013). Quality of life in head and neck cancer patients receiving multimodal or targeted therapy: Update of the EORTC QLQ-H&N35 Phase I. Head and Neck.

[21] Singer S, Arraras J, Chie WC, Fisher S, Galalae R, Hammerlid E (2013). Performance of the EORTC questionnaire for the assessment of quality of life in head and neck cancer patients EORTC QLQ-H&N35. A methodological review. Quality of Life Research.

[22] Bjordal K, Ahlner-Elmqvist M, Hammerlid E, Boysen M, Evensen JF, Biorklund A (2001). A prospective study of quality of life in head and neck cancer patients Part II: Longitudinal Data. The Laryngoscope.

[23] Nordgren M, Hammerlid E, Bjordal K, Ahlner-Elmqvist M, Boysen M, Jannert M (2008). Quality of life in oral carcinoma: A 5-year prospective study. Head & Neck.

[24] Abendstein H, Nordgren M, Boysen M, Jannert M, Silander EM, Ahlner-Elmqvist M, Hammerlid E, Bjordal K (2005). Quality of life and head and neck cancer: A 5 year prospective study. The Laryngoscope.

[25] Roick J, Danker H, Dietz A, Papsdorf K, Singer S (2020). Predictors of changes in quality of life in head and neck cancer patients: A prospective study over a six-month period. European Archives of Oto-Rhino-Laryngology.

[26] Taylor K, Singer S (2019). Long-term quality of life in head and neck cancer patients A systematic review. Der Onkologe.

[27] Singer S, Danker H, Guntinas-Lichius O, Oeken J, Pabst F, Schock J, Vogel HJ, Meister EF, Wulke C, Dietz A (2014). Quality of life before and after total laryngectomy: Results of a multi-centre prospective cohort study. Head and Neck.

[28] Aaronson N, Ahmedzai S, Bergmann B, Bullinger M, Cull A, Duez NJ (1993). The European Organization for Research and Treatment of Cancer QLQ-C30: A Quality-of-Life Instrument for use in international clinical trials in oncology. Journal of the National Cancer Institute.

[29] Fayers PM, Machin D (2007). Quality of Life.

[30] Devji T, Carrasco-Labra A, Qasim A, Phillips M, Johnston BC, Devasenapathy N, Zeraatkar D, Bhatt M, Jin X, Brignardello-Petersen R, Urquhart O (2020). Evaluating the credibility of anchor based estimates of minimal important differences for patient reported outcomes: instrument development and reliability study. British Medical Journal.

[31] Youden WJ (1950). Index for rating diagnostic tests. Cancer.

[32] Terluin B, Eekhout I, Terwee C, De Vet HCW (2015). Minimal important chnge (MIC) based on a predictive modeling approach was more precise than MIC based on ROC analysis. Journal of Clinical Epidemiology.

[33] Crary MA, Mann GDC, Groher ME (2005). Initial psychometric assessment of a functional oral intake scale for dysphagia in stroke patients. Archives of Physical Medicine and Rehabilitation.

[34] Rosenbek JC, Robbins JA, Roecker EB, Coyle JL, Wood JL (1996). A penetration aspiration scale. Dysphagia.

[35] Hutcheson KA, Barrow MP, Barringer DA, Knott JK, Lin HY, Weber RS (2017). Dynamic imaging grade of swallowing toxicity (DIGEST): Scale development and validation. Cancer.

[36] Chen AY, Frankowski R, Bishop-Leone J, Hebert T, Leyk S, Lewin J (2001). The development and validation of a dysphagia-specific quality-of-life questionnaire for patients with head and neck cancer: The M. D Anderson dysphagia inventory. Archives of Otolaryngology-Head & Neck Surgery.

[37] Carnaby GD, Crary MA (2014). Development and validation of a cancer-specific swallowing assessment tool: MASA-C. Supportive Care in Cancer.

[38] Cocks K, Velikova G, King MT, Fayers PM, Brown JM (2014). Can individual patients assess differences in quality of life between groups of patients?. European Journal of Cancer Care.

[39] Bindewald, J., Herrmann, E., Dietz, A., Wulke, C., Meister, E. F., Wollbruck, D. et al. (2007). Quality of life and voice intelligibility in laryngeal cancer patients: Relevance of the "satisfaction paradox". [German], *Laryngo- Rhino- Otologie* 86, 426-430 10.1055/s-2007-966167 17654777

[40] Schwartz CE, Bode R, Repucci N, Becker J, Sprangers MA, Fayers P (2006). The clinical significance of adaptation to changing health: A meta-analysis of response shift. Quality of Life Research.

[41] Sprangers MA, Schwartz CE (1999). Integrating response shift into health-related quality of life research: A theoretical model. Social Science and Medicine.

[42] Giesinger JM, Aaronson NK, Arraras JI, Efficace F, Groenvold M, Kieffer JM (2018). A cross-cultural convergent parallel mixed methods study of what makes a cancer-related symptom or functional health problem clinically important. Psycho-Oncology.

[43] Fahsl S, Keszte J, Boehm A, Vogel H-J, Völkel W, Meister EF (2012). Clinical relevance of quality of life data in laryngectomized patients. The Laryngoscope.

