# Supplementary material for: Methodological approach for determining the Minimal Important Difference and Minimal Important Change scores for the European Organisation for Research and Treatment of Cancer Head and Neck Cancer Module (EORTC QLQ-HN43) exemplified by the Swallowing scale
Source: Qual Life Res. 2021 Jul 16;31(3):841–53. doi: 10.1007/s11136-021-02939-6 (PMC8921167; doi:10.1007/s11136-021-02939-6)
Supplement: Supplementary file 1 — (DOCX 508 kb) [file 11136_2021_2939_MOESM1_ESM.docx]

# Supplemental material

eTable 1: Correlation between Karnofsky Performance Score (KPS) and EORTC QLQ-HN43 scales

| **Scales of the EORTC QLQ-HN43** | **Spearman rank correlation coefficient** | |
| --- | --- | --- |
|  | **at t1** | **at t2** |
| Social contact (SC) | -0.34 | -0.42 |
| Social eating (SO) | -0.38 | -0.42 |
| Body image (BI) | -0.30 | -0.39 |
| Speech (SP) | -0.31 | -0.39 |
| Swallowing (SW) | -0.32 | -0.36 |
| Problems with teeth (TH) | -0.32 | -0.33 |
| Fear of progression (AX) | -0.20 | -0.32 |
| Problems with senses (SE) | -0.30 | -0.29 |
| Pain in the mouth (PA) | -0.16 | -0.27 |
| Swelling in the neck (SN) | -0.08 | -0.27 |
| Weight loss (WL) | -0.19 | -0.26 |
| Problems opening mouth (OM) | -0.18 | -0.25 |
| Dry mouth and sticky saliva (DR&SS) | -0.22 | -0.24 |
| Skin problems (SK) | -0.16 | -0.24 |
| Problems with wound healing (WO) | -0.16 | -0.24 |
| Coughing (CO) | -0.23 | -0.22 |
| Sexuality (SX) | -0.17 | -0.18 |
| Problems with shoulder (SH) | -0.18 | -0.17 |
| Neurological problems (NE) | -0.13 | -0.12 |

eTable 2: Detailed report of sensitivity and specificity per cut point in changes in swallowing problems between t1 and t2 detecting deterioration

| **Cutpoint >=** | **Sensitivity** | **Specificity** | **Youden-Index** |
| --- | --- | --- | --- |
| -100 | 1.000 | 0.000 | 0.000 |
| -79 | 0.990 | 0.000 | -0.010 |
| -54 | 0.980 | 0.000 | -0.020 |
| -46 | 0.970 | 0.027 | -0.003 |
| -38 | 0.970 | 0.036 | 0.006 |
| -33 | 0.960 | 0.054 | 0.014 |
| -29 | 0.960 | 0.090 | 0.050 |
| -25 | 0.949 | 0.090 | 0.040 |
| -25 | 0.949 | 0.108 | 0.058 |
| -21 | 0.949 | 0.117 | 0.067 |
| -17 | 0.939 | 0.171 | 0.111 |
| -17 | 0.929 | 0.189 | 0.118 |
| -12 | 0.929 | 0.198 | 0.127 |
| -8 | 0.919 | 0.279 | 0.198 |
| -4 | 0.909 | 0.324 | 0.233 |
| 4 | 0.788 | 0.604 | 0.391 |
| 8 | 0.768 | 0.613 | 0.380 |
| 8 | 0.707 | 0.703 | 0.410 |
| 8 | 0.697 | 0.703 | 0.400 |
| 11 | 0.657 | 0.703 | 0.359 |
| 15 | 0.646 | 0.703 | 0.349 |
| 17 | 0.636 | 0.712 | 0.348 |
| 17 | 0.556 | 0.766 | 0.321 |
| 21 | 0.545 | 0.784 | 0.329 |
| 25 | 0.424 | 0.847 | 0.271 |
| 29 | 0.414 | 0.847 | 0.261 |
| 33 | 0.354 | 0.865 | 0.218 |
| 38 | 0.323 | 0.865 | 0.188 |
| 42 | 0.323 | 0.874 | 0.197 |
| 46 | 0.253 | 0.937 | 0.189 |
| 53 | 0.192 | 0.946 | 0.138 |
| 57 | 0.192 | 0.955 | 0.147 |
| 58 | 0.141 | 0.991 | 0.132 |
| 63 | 0.131 | 0.991 | 0.122 |
| 71 | 0.101 | 0.991 | 0.092 |
| 76 | 0.051 | 0.991 | 0.041 |
| 81 | 0.040 | 0.991 | 0.031 |
| 88 | 0.030 | 1.000 | 0.030 |
| 96 | 0.020 | 1.000 | 0.020 |
| 100 | 0.000 | 1.000 | 0.000 |

eTable 3: Detailed report of sensitivity and specificity per cutpoint in changes in swallowing problems between t2 and t3 detecting improvement

| **Cutpoint >=** | **Sensitivity** | **Specificity** | **Youden-Index** |
| --- | --- | --- | --- |
| -84 | 1.000 | 0.000 | 0.000 |
| -83 | 0.990 | 0.000 | -0.010 |
| -71 | 0.980 | 0.000 | -0.020 |
| -58 | 0.961 | 0.000 | -0.039 |
| -54 | 0.951 | 0.011 | -0.038 |
| -50 | 0.931 | 0.032 | -0.036 |
| -46 | 0.922 | 0.032 | -0.046 |
| -42 | 0.912 | 0.043 | -0.045 |
| -38 | 0.863 | 0.043 | -0.094 |
| -33 | 0.833 | 0.054 | -0.113 |
| -29 | 0.765 | 0.097 | -0.139 |
| -25 | 0.755 | 0.097 | -0.148 |
| -25 | 0.735 | 0.108 | -0.157 |
| -21 | 0.627 | 0.129 | -0.244 |
| -17 | 0.588 | 0.151 | -0.261 |
| -13 | 0.529 | 0.151 | -0.320 |
| -8 | 0.500 | 0.161 | -0.339 |
| -8 | 0.461 | 0.172 | -0.367 |
| -8 | 0.382 | 0.204 | -0.413 |
| -4 | 0.363 | 0.269 | -0.368 |
| 3 | 0.157 | 0.656 | -0.187 |
| 7 | 0.157 | 0.667 | -0.176 |
| 8 | 0.147 | 0.677 | -0.176 |
| 8 | 0.108 | 0.710 | -0.182 |
| 8 | 0.098 | 0.720 | -0.182 |
| 11 | 0.098 | 0.753 | -0.149 |
| 15 | 0.088 | 0.753 | -0.159 |
| 17 | 0.088 | 0.763 | -0.148 |
| 17 | 0.078 | 0.785 | -0.137 |
| 17 | 0.059 | 0.806 | -0.135 |
| 21 | 0.049 | 0.806 | -0.145 |
| 25 | 0.039 | 0.806 | -0.154 |
| 25 | 0.020 | 0.849 | -0.131 |
| 29 | 0.020 | 0.860 | -0.120 |
| 33 | 0.010 | 0.892 | -0.098 |
| 38 | 0.010 | 0.914 | -0.076 |
| 43 | 0.000 | 0.914 | -0.086 |
| 47 | 0.000 | 0.925 | -0.075 |
| 54 | 0.000 | 0.935 | -0.065 |
| 62 | 0.000 | 0.946 | -0.054 |

eFigure 1: Sensitivity and specificity at various cut-points of swallowing change scores measured with the EORTC QLQ-HN43 in relation to "swallowing deteriorated/improved" from the patient perspective, measured with the Subjective Significance Questionnaire

### Panel a: for deterioration (change from t1 to t2)

### Panel b: for improvement (change from t2 to t3)
